# Target Metabolites to Slow Down Progression of Amyotrophic Lateral Sclerosis in Mice

**DOI:** 10.3390/metabo12121253

**Published:** 2022-12-12

**Authors:** Destiny Ogbu, Yongguo Zhang, Katerina Claud, Yinglin Xia, Jun Sun

**Affiliations:** 1 Department of Medicine, University of Illinois Chicago, Chicago, IL 60612, USA; 2 Department of Microbiology/Immunology, University of Illinois Chicago, Chicago, IL 60612, USA; 3 Jesse Brown VA Medical Center, Chicago, IL 60612, USA

**Keywords:** ALS, CNS, dysbiosis, FALS, inflammation, longitudinal analysis, immunity, metabolites, neuromuscular disease, protein aggregation, SALS

## Abstract

Microbial metabolites affect the neuron system and muscle cell functions. Amyotrophic lateral sclerosis (ALS) is a multifactorial neuromuscular disease. Our previous study has demonstrated elevated intestinal inflammation and dysfunction of the microbiome in patients with ALS and an ALS mouse model (human-SOD1^G93A^ transgenic mice). However, the metabolites in ALS progression are unknown. Using an unbiased global metabolomic measurement and targeted measurement, we investigated the longitudinal changes of fecal metabolites in SOD1^G93A^ mice over the course of 13 weeks. We further compared the changes of metabolites and inflammatory response in age-matched wild-type (WT) and SOD1^G93A^ mice treated with the bacterial product butyrate. We found changes in carbohydrate levels, amino acid metabolism, and the formation of gamma-glutamyl amino acids. Shifts in several microbially contributed catabolites of aromatic amino acids agree with butyrate-induced changes in the composition of the gut microbiome. Declines in gamma-glutamyl amino acids in feces may stem from differential expression of gamma-glutamyltransferase (GGT) in response to butyrate administration. Due to the signaling nature of amino acid-derived metabolites, these changes indicate changes in inflammation, e.g., histamine, and contribute to differences in systemic levels of neurotransmitters, e.g., γ-Aminobutyric acid (GABA) and glutamate. Butyrate treatment was able to restore some of the healthy metabolites in ALS mice. Moreover, microglia in the spinal cord were measured by IBA1 staining. Butyrate treatment significantly suppressed the IBA1 level in the SOD1^G93A^ mice. Serum IL-17 and LPS were significantly reduced in the butyrate-treated SOD1^G93A^ mice. We have demonstrated an inter-organ communications link among microbial metabolites, neuroactive metabolites from the gut, and inflammation in ALS progression. The study supports the potential to use metabolites as ALS hallmarks and for treatment.

## 1. Introduction

Amyotrophic lateral sclerosis (ALS), also known as Lou Gehrig’s disease, is a multifactorial neurodegenerative disease characterized by the premature death of motor neurons and has an average survival of three to five years in humans [1]. Currently, treatment with the FDA-approved drug Riluzole merely extends the patient’s lifespan for a few months [2]. A second drug, Radicava (Edavarone), was approved in 2017 and shows moderate efficacy [3]. On September 29, 2022, the Food and Drug Administration approved Relyvrio for ALS treatment. Relyvrio is a combination of sodium phenylbutyrate and taurursodiol, which was shown to reduce the rate of decline on a clinical assessment of daily functioning and was associated with longer overall survival. We have shown the beneficial role of the bacterial metabolite butyrate in treating dysbiosis, intestinal leakage, and abnormal function of enteric neurons in the SOD1^G93A^ model [4,5]. Better understanding the pathological changes in ALS will facilitate new treatments for alleviating disease progression and improving the life quality of patients with ALS.

Inter-organ communications may play important role in the disease progression. Celiac disease with neurologic manifestations has been misdiagnosed as ALS [6,7,8]. There is a possible link between ALS and sensitivity to gluten [9]. Moreover, inflammatory cytokines and bacterial product lipopolysaccharides (LPS) have been found to be elevated in ALS [10,11]. Autoimmune disease (e.g., Crohn’s disease) associations with ALS indicate the contribution of genetic or environmental risk factors to the pathogenesis and progression of ALS [12]. Early diagnosis of ALS has been a long-standing challenge in the field. Researchers have explored metabolomics as potential diagnostic markers for ALS. Human and mouse studies have shown alterations in carbohydrate, amino acid, and lipid metabolism within the serum and cerebral spinal fluid [13,14,15]. It is reported that altered metabolites in the ALS-like SOD1^G93A^ model are associated with a progressive state of acidosis [16]. Thus, understanding the metabolomics and intestinal changes will be important for the neuron pathogenesis in ALS.

Research of metabolism in ALS through the microbiome is a growing field. We were the first to report elevated intestinal inflammation/permeability and reduced beneficial bacteria in human ALS [17]. Furthermore, we reported that the bacterial metabolite butyrate reduced dysbiosis and restored functions of the intestinal barrier and enteric neurons in the SOD1^G93A^ model [4,5]. The metabolite nicotinamide is reported to potentially contribute to the ALS disease phenotype [18]. In a small study that compared patients with household controls, the ALS group showed reduced nicotinamide systemically and in the cerebrospinal fluid [18]. These findings suggest that metabolites may drive the ALS pathology through the gut–neuron–microbiome axis. However, studies focusing on the microbial metabolites in ALS as well as the metabolic changes following butyrate treatment are lacking. 

The goal of this study is to characterize metabolic changes in the SOD1^G93A^ model and to gain insights into the fundamentals of neuromuscular function and metabolites in ALS treatment by targeting metabolites. The transgenic mouse model SOD1^G93A^ was chosen for this study because a subset of genetic or familial ALS (fALS) is associated with mutations in the Cu/Zn superoxide dismutase 1 gene, or *SOD1*. Furthermore, the SOD1^G93A^ model shows neuronal and muscular impairment like human ALS [19]. In addition, the preclinical model exhibits defective metabolism in tissues like skeletal muscle to major energetic pathways and whole-body energy alterations [20]. The whole-body alterations suggest an inter-organ communication. Moreover, we examined the changes of metabolites from feces of SOD1^G93A^ mice with or without butyrate treatment and correlated them with inflammation and neurological changes. Our data suggest that changes within the metabolite profile are correlated with altered inflammatory cytokines and disease progression. Importantly, butyrate treatment significantly reduced metabolic differences between the wild-type (WT) and SOD1^G93A^ mice. Better understanding the intestinal dysfunction and metabolites in ALS will help in early diagnosis and the development of new treatments. 

## 2. Materials and Methods

### 2.1. Animals

SOD1^G93A^ and age-matched WT mice were used in this study. SOD1^G93A^ mice were originally purchased from Jackson Laboratory (Bar Harbor, ME, USA) (B6SJL-Tg (SOD1-G93A) 1Gur/J, stock No. 002726). Mice were bred and housed at the Animal Facility at UIC. The mice were provided with water ad libitum and maintained in a room with a 12 h dark/light cycle. Multiple breeding pairs were set up within a specific vivarium room where the environment, cage changes, and dietary schedules are more uniform. The untreated mice cohort included 10 mice, where 5 were wild-type and 5 were SOD1^G93A^ (each group consisted of 3 males and 2 females). The butyrate-treated group consisted of 12 mice in total, where 6 were wild-type and 6 were SOD1^G93A^ (each group included 3 males and 3 females). All experiments were carried out in strict accordance with the recommendations in the *Guide for the Care and Use of Laboratory Animals* of the National Institutes of Health. All animal work adhered to the ARRIVE guidelines. The protocol was approved by the IACUC of the University of Illinois Chicago Committee on Animal Resources (ACC 18-233). 

### 2.2. Butyrate Treatment in Mice

SOD1^G93A^ mice and age-matched WT mice were divided into two groups randomly: the non-treatment (control) group and the butyrate treatment group. The butyrate-treated group received sodium butyrate (Sigma-Aldrich, 303410, St. Louis, MO, USA) at a 2% concentration in filtered drinking water. The control group received filtered drinking water without sodium butyrate. All animals were weighted and received a detailed clinical examination, which included appearance, movement and behavior patterns, skin and hair conditions, eyes, and mucous membranes. If restricted outstretching of the hind legs was observed when the tail was held, this is an obvious symptom of ALS. After a mouse is laid on its back, if it cannot turn over in 30 s, the mouse is humanely sacrificed.

### 2.3. Fecal Sample Collection

We collected fecal samples from 6 WT and 6 SOD1^G93A^ mice at baseline (mice age: 9 weeks), 3 weeks after butyrate treatment (mice age: 12 weeks), and 6 weeks after butyrate treatment (mice age: 15 weeks). For untreated animals, fecal samples were collected from 5 WT and 5 SOD1^G93A^ mice (at 4, 8, 13, and 17 weeks old). Global metabolic profiles were determined by Metabolon.

### 2.4. Bioinformatic Analysis of Metabolomics Data via Metabolon Platform

#### 2.4.1. Sample Accessioning and Sample Preparation

Fecal sample accessioning and sample preparation were processed via the Metabolon LIMS system. Following receipt, all fecal samples were inventoried and immediately stored at −80 °C until processing. Samples were prepared using the automated MicroLab STAR^®^ system from Hamilton Company (Reno, NV, USA). Before processing extraction, for quality control (QC) and quality assurance (QA), several recovery standards were added to remove protein, dissociate small molecules bound to protein or trapped in the precipitated protein matrix, and to recover chemically diverse metabolites, and proteins were precipitated with methanol under vigorous shaking for 2 min (Glen Mills GenoGrinder 2000. Clifton, NJ, USA) followed by centrifugation. The resulting extract was divided into five fractions: two for separate analyses by the reverse phase [21] and ultrahigh performance liquid chromatography–tandem mass spectroscopy (UPLC-MS/MS) methods with positive ion mode electrospray ionization (ESI) [18], one for analysis by RP/UPLC-MS/MS with negative ion mode ESI, one for analysis by HILIC/UPLC-MS/MS with negative ion mode ESI, and one reserved for backup. Samples were placed briefly on a TurboVap^®^ (Zymark, Hopkinton, MA, USA) to remove the organic solvent. The sample extracts were stored overnight under nitrogen before preparation for analysis.

#### 2.4.2. QA/QC

First, several types of controls were analyzed in concert with the experimental samples based on the Metabolon QC recovery and internal standards, including: (1) a pooled matrix sample generated by taking a small volume of each experimental sample to serve as a technical replicate throughout the data set; (2) extracted water samples to serve as process blanks; and (3) a carefully chosen cocktail of QC standards to ensure it does not interfere with the measurement of endogenous compounds and spiked into every analyzed sample, allowing instrument performance monitoring and aiding chromatographic alignment. Then, instrument variability was determined by calculating the median relative standard deviation (RSD) for the standards that were added to each sample prior to injection into the mass spectrometers. Overall process variability was determined by calculating the median RSD for all endogenous metabolites (i.e., non-instrument standards) present in 100% of the pooled matrix samples. Experimental samples were randomized across the platform run with QC samples spaced evenly among the injections, as outlined in Figure S1. 

#### 2.4.3. Data Extraction and Compound Identification

Raw metabolomics data were generated by the UPLC-MS/MS methods. All methods utilized a Waters ACQUITY ultra-performance liquid chromatography (UPLC) system and a Thermo Scientific Q-Exactive high resolution/accurate mass spectrometer interfaced with a heated electrospray ionization (HESI-II) source and Orbitrap mass analyzer operated at 35,000 mass resolution. The sample extract was dried and then reconstituted in solvents compatible with each of the four methods as described in sample preparation. Raw data were extracted, peak-identified, and QC processed using Metabolon’s hardware and software. Compounds were identified by comparison to library entries of more than 3300 commercially available purified standard compounds or recurrent unknown entities. Metabolon maintains a library based on authenticated standards that contains the retention time/index (RI), mass-to-charge ratio (*m*/*z*), and chromatographic data (including MS/MS spectral data) on all molecules present in the library. Thus, biochemical identifications are based on three criteria: (1) retention index within a narrow RI window of the proposed identification, (2) accurate mass match to the library, +/−10 ppm, and (3) the MS/MS forward and reverse scores between the experimental data and authentic standards. The MS/MS scores are based on a comparison of the ions present in the experimental spectrum to the ions present in the library spectrum. While there may be similarities between the molecules based on one of these factors, the use of all three data points can be utilized to distinguish and differentiate biochemicals. 

#### 2.4.4. Curation

To ensure the available data set for statistical analysis and data interpretation has a high quality, a variety of QC and curation procedures were carried out to ensure accurate and consistent identification of true chemical entities, and to remove those representing system artifacts, mis-assignments, and background noise. For example, Metabolon data analysis was performed to confirm the consistency of peak identification among the various samples using proprietary visualization and interpretation software. Library matches for each compound were checked for each sample and corrected if necessary.

#### 2.4.5. Metabolite Quantification and Data Normalization

Peaks were quantified using the area-under-the-curve method. For butyrate metabolites, quantitative short-chain fatty acid panels were utilized for accurate distinction between isomer metabolites and for quantification of butyrate in fecal samples. Data normalization was performed based on whether the studies required more than one day of analysis or not. No normalization was performed for those studies that required one day of analysis. For studies spanning multiple days, a data normalization step was performed to correct variations resulting from instrument inter-day tuning differences. Essentially, each compound was corrected in run-day blocks by registering the medians to equal one (1.00) and normalizing each data point proportionately (termed the “block correction”). In certain instances, biochemical data may have been normalized to an additional factor (e.g., cell counts, total protein as determined by Bradford assay, osmolality, etc.) to account for differences in metabolite levels due to differences in the amount of material present in each sample.

#### 2.4.6. Immunofluorescence

Lumbar spine tissues were freshly isolated and paraffin-embedded after fixation with 10% neutral buffered formalin. Immunofluorescence was performed on paraffin-embedded sections (5 μm). After preparation of the slides as described previously [22], tissue samples were incubated with anti-IBA1 antibody (Cell Signaling, 17198, Danvers, MA, USA) at 4 °C overnight. Samples were then incubated with goat anti-rabbit Alexa Flour 594 (Invitrogen, A32740, Carlsbad, CA, USA) for 1 h at room temperature. Tissues were mounted with Slow Fade (Invitrogen, s2828, Carlsbad, CA, USA), followed by a coverslip, and the edges were sealed to prevent drying. Specimens were examined with Zeiss laser scanning microscope (LSM) 710. Fluorescence intensity was determined by using Image J software, Image J Version Fiji, https://imagej.net/software/fiji/, accessed on 10 November 2022). This method determines the corrected total fluorescence by subtracting out background signals, which is useful for comparing the fluorescence intensity between cells or regions.

#### 2.4.7. Mouse Cytokines

Mouse blood samples were collected by cardiac puncture and placed in tubes containing EDTA (10 mg/mL). Mouse cytokines were measured using a Cytokine & Chemokine Convenience 26-Plex Mouse ProcartaPlex™ Panel 1 (Invitrogen, EPXR260-26088-90, Carlsbad, CA, USA) according to the manufacturer’s instructions. Briefly, beads of defined spectral properties were conjugated to protein-specific capture antibodies and added along with samples (including standards of known protein concentration, control samples, and test samples) into the wells of a filter-bottom microplate, where proteins bound to the capture antibodies over the course of a 2 h incubation. After washing the beads, protein-specific biotinylated detector antibodies were added and incubated with the beads for 1 h. After the removal of excess biotinylated detector antibodies, the streptavidin-conjugated fluorescent protein R-phycoerythrin was added and allowed to incubate for 30 min. After washing to remove unbound streptavidin–R-phycoerythrin, the beads were analyzed with a Luminex detection system (Bio-rad, Bio-Plex 200 Systems, Hercules, CA, USA).

#### 2.4.8. LPS Detection

LPS in serum samples was measured with limulus amebocyte lysate chromogenic end point assays (Hycult Biotech, HIT302, Plymouth, PA, USA) according to the manufacturer’s indications. The samples were diluted at a 1:4 ratio with endotoxin-free water and then heated at 75 °C for 5 min on a warm plate to denature the protein before the reaction. A standard curve was generated and used to calculate the concentrations, which were expressed as EU/mL, in the serum samples.

#### 2.4.9. Statistical Analysis

Generally, all the data except metabolites are expressed as the means ± SD. All statistical tests were 2-sided. The *p*-values ≤ 0.05 were considered statistically significant. The significant *p*-values were labeled in the figures. To ensure the accuracy of the statistical significance judgments, the adjusted *p*-values (*q*-values) for correction of multiple comparisons were also provided in the figure legends accordingly. For metabolite data, a fold-change ratio of genotype vs. control was used to compare the differences between the G93A and WT groups, and a fold-change ratio between time points as indicated within the G93A or WT group was used to compare the differences over time points for the G93A and WT groups. For standardized metabolite data, a Welch’s two-sample *t*-test was utilized to assess the mean differences of fold-change values between the study groups. To accommodate high within-group variability, a non-parametric Wilcoxon rank sum test was also performed to compare the median differences of fold-change values between the study groups. In this study, the Wilcoxon rank sum test returned results similar to those of Welch’s two-sample *t*-test. Thus, the biochemical interpretation of this study was based on the results of the Welch’s two-sample *t*-test. Principal component analysis (PCA) was performed to reduce the dimensionality of the data for visually assessing similarities and differences between samples. Random forest (RF) analysis was performed to identify the biomarkers differentiating classification groups. RF analysis generated an accompanying list of the top metabolites contributing most to the separation of the groups being compared. 

For all the experiment data not from the Metabolon platform, the differences between samples for more than two groups were analyzed using one-way ANOVA or its non-parametric alternative, the Kruskal–Wallis test, based on the data normality or non-detection by the Shapiro–Wilks normality test. The *p*-values in ANOVA analyses were adjusted for the correction of multiple comparisons using the Tukey method to ensure accurate results. All the metabolomics data were analyzed via the Metabolon platform. All the other statistical analyses except for metabolite data were performed using either the GraphPad Prism 9.2.0 software (GraphPad, Inc., San Diego, CA, USA) or the R software (R version 4.0.4, 2021, The R Foundation for Statistical Computing Platform). 

## 3. Results

### 3.1. Altered Microbial Metabolites in the ALS Mice over Course of Disease

**Figure 1:**
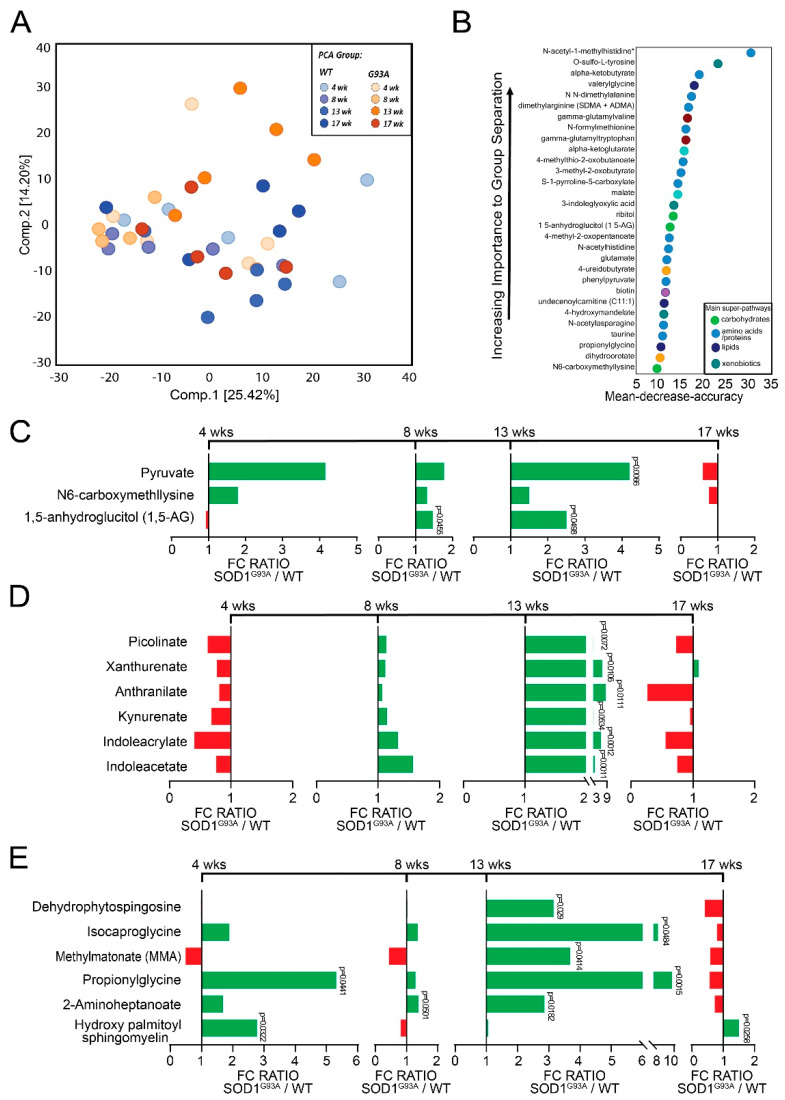
Impact of butyrate treatment on metabolite profile in ALS mice over the course of the disease. (**A**) PCA comparing the biochemical similarity between WT and SOD1^G93A^ mice over the course of disease. (**B**) Random forest (RF) analysis showing the top 30 most important metabolites that differed between WT and SOD1^G93A^ mice over the course of disease. The variables are ordered top to bottom as most to least important. Different colors indicate specific metabolites resulting from different superpathways: green = carbohydrate, light blue = amino acid, blue = lipids, and teal = xenobiotics. Biochemical Name * indicates compounds that have not been officially confirmed based on a standard, but the identity was confirmed by Metabolon (Morrisville, NC, USA). (**C**–**E**) Welch’s *t*-tests are used to compare the fold change ratios of the average concentrations of metabolites between SOD1^G93A^ and WT at 4, 8, 13, and 17 weeks. Metabolites are categorized as: (**C**) carbohydrate, (**D**) amino acid, or (**E**) lipid. Fold-change ratios at 4; 8; 13; and 17 weeks generated by (**C**) carbohydrate (at week 13, pyruvate for *q* = 0.0797, 1,5-anhydroglucitol for *q* = 0.1313); (**D**) amino acid (at week 13 picolinate, *q* = 0.1281; for xanthurenate, *q* = 0.0879; for anthranilate, *q* = 0.0879; for kynurenate, *q* = 0.1350; for indoleacrylate, *q* = 0.0532; for indoleacetate, *q* = 0.0532); (**E**) lipid (at week 4 for propionylglycine, *q* = 0.632; for hydroxypalmitoyl sphingomyelin, *q* = 0.632. At week 8 for 2-aminoheptanoate, *q* = 0.8946. At week 13 for dehydrophytosphingosine, *q* = 0.1117; isocaprolylglycine, *q* = 0.1298; methylmalonate, *q* = 0.1298; for Propionylglycine, *q* = 0.0532; for 2-Aminoheptanoate, *q* = 0.1058. At week 17, for hydroxypalmitoyl sphingomyelin *q* = 0.9941) changes over time. Differences are assessed by the Welch’s two-sample *t*-test. WT (N = 5) and SOD1^G93A^ (N = 5) mice. The *p*-values and *q*-values are listed for 4; 8; 13; and 17 weeks.

ALS is a progressive disorder, where the onset of muscle weakness spreads to adjacent body regions [23]; thus, we evaluated when the fecal metabolite profile changed over the course of the disease. We investigated the longitudinal changes of fecal metabolites in ALS progression using SOD1^G93A^ mice at 4, 8, 13, and 17 weeks old. There is a total of 797 compounds identified in the samples. We utilized a principal component analysis (PCA) to visually compare the metabolic profile at 4, 8, 13, and 17 weeks old between untreated WT and SOD1^G93A^ mice. The PCA did not show sample separation related to time or genotype, which may be attributed to inter-sample variability in masking the effects related to the genotype. However, regardless of the overlapping between data samples, PCA showed that individual metabolite levels still explain 39.62 % (25.42 % + 14.20%) of the group separation over time, and the difference was particularly visible at week 13 (Figure 1A).

As shown in Table 1, SOD1^G93A^ mice experience changes in a total of 258 biochemicals with significance of *p* ≤ 0.05 from 4 weeks to 17 weeks of age. We compared the changes in metabolites using a ratio of SOD1^G93A^ to WT mice via Welch’s two-sample *t*-test. At 4 weeks, 61 metabolites were altered (of which 49 increased and 12 decreased). At 8 weeks, seven metabolites changed (of which five increased and two decreased). SOD1^G93A^ mice had a total of 180 metabolites (of which 175 increased while 5 decreased) change at 13 weeks. Lastly, 10 metabolites changed for SOD1^G93A^ mice at 17 weeks (of which 6 increased and 4 decreased). Thus, these observations suggest significant changes of metabolites in the progress of the disease in the SOD1^G93A^ mice. 

Next, we observed an RF analysis of metabolites between WT and SOD1^G93A^ animals which revealed the impact of different metabolites (Figure 1B). A list was made of 30 biochemicals contributing to the difference between WT and SOD1^G93A^ mice. The top metabolite groups are amino acids/proteins and xenobiotics. The most significant alterations in metabolites were generated by four super-pathways: (A) carbohydrates, (B) amino acids /proteins, (C) lipids, and (D) xenobiotics metabolism. The top three metabolites were N-acetyl-1-methylhistidine, alpha-ketobutyrate, and O-sulfo-L-tyrosine, which were in the amino acids/protein and xenobiotics groups, respectively.

#### 3.1.1. Changes in Carbohydrates in the SOD1^G93A^ Mice

Carbohydrate fermentation yields dietary fibers that are a primary source of energy for gut microbiota [24]. Defects in glucose metabolism may contribute to disease progression in ALS [25]. Figure 1C shows changes in some glucose-related carbohydrate metabolites for SOD1^G93A^ mice over the disease course. SOD1^G93A^ mice had a significant increase in 1,5-anhydroglucitol (1,5-AG) at 8 weeks. At 13 weeks of age, 1,5-anhydroglucitol and pyruvate significantly increased. Fecal metabolite levels of glucose-related carbohydrates differed between ALS and WT mice. 

#### 3.1.2. Changes of Amino Acids/Proteins in SOD1^G93A^ Mice

Tryptophan is an essential amino acid in the microbiome [26], and metabolism of tryptophan is altered in ALS pathogenesis [27]. We observed changes in tryptophan metabolism in ALS mice over the disease course (Figure 1D). The tryptophan-related metabolites xanthurenate, anthranilate, kynurenate, indoleacrylate, and indoleacetate significantly increased at 13 weeks for SOD1^G93A^ mice. 

#### 3.1.3. Changes in Lipids in SOD1^G93A^ Mice

Dysfunctions in lipid metabolism, including fatty acids, triacylglycerols, phospholipids, sterol lipids, and sphingolipids, have been identified as potential drivers of pathogenesis in ALS [28]. Figure 1E shows observed changes in sphingomyelins, sphingosines, and fatty acids in SOD1^G93A^ mice. At 4 weeks of age, SOD1^G93A^ mice had increases in propionylglycine and hydroxy palmitoyl sphingomyelin; 2-aminoheptanoate increased at 8 weeks of age. At 13 weeks of age, dehydrophytospingosine, isocaproglycine, methylmatonate (MMA), propionylglycine, and 2-aminoheptanoate significantly increased. Only hydroxy palmitoyl sphingomyelin increased at 17 weeks of age for SOD1^G93A^ mice. These results depict changes in lipid metabolism during ALS progression. 

### 3.2. Dietary Butyrate Treatment Altered the Metabolite Profile of ALS Mice over Disease Course 

**Figure 2:**
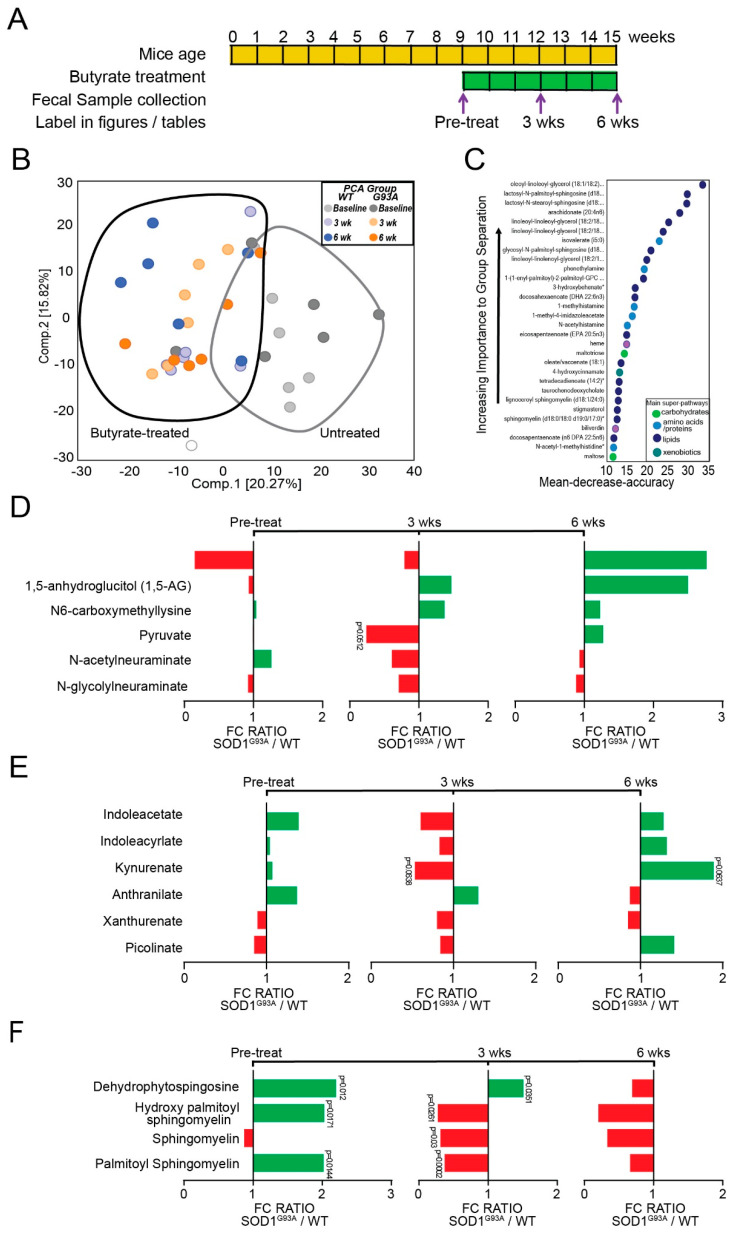
Effect of butyrate treatment on metabolite profile in ALS mice over the course of disease. (**A**) Schematic overview of the WT/SOD1^G93A^ mice treated with butyrate. Male or female SOD1^G93A^ mice were treated with or without 2% butyrate in drinking water starting with mice aged 9 weeks to 15 weeks. Fecal samples were harvested pre-treatment (mice age: 9 weeks), 3 weeks after butyrate treatment (mice age: 12 weeks), and 6 weeks (mice age: 15 weeks) after butyrate treatment. (**B**) PCA comparing the biochemical similarity between butyrate-treated WT and SOD1^G93A^ mice. (**C**) RF showing the top 30 most important metabolites that differed between longitudinal butyrate-treated WT and SOD1^G93A^ mice. The *p*-values and *q*-values are listed for pre-treatment; 3 weeks after butyrate treatment; and 6 weeks after butyrate treatment. Biochemical Name *: indicates compounds that have not been officially confirmed based on a standard, but the identity was confirmed by Metabolon (Morrisville, NC, USA).(**D**–**F**) Welch’s *t*-tests are used to compare the fold-change ratios of the average concentrations of metabolites between SOD1^G93A^ and WT pre-treatment, 3 weeks after butyrate treatment, and 6 weeks after butyrate treatment. Metabolites are categorized as: (**D**) carbohydrate, (**E**) amino acid, or (**F**) Lipid. Fold-change ratios generated by (**D**) carbohydrates (3 weeks after butyrate treatment, for pyruvate, *q* = 0.2546) (**E**) amino acids (3 weeks after butyrate treatment, for kynurenate, *q* = 0.2728; 6 weeks post butyrate treatment, for kynurenate, *q* = 0.3172) (**F**) lipid (pre-treatment, for dehydrophytosphingosine, *q* = 0.2687; for hydroxypalimotyl sphingomyelin, *q* = 0.2687; for palimitoyl sphingomyelin, *q* = 0.2687. 3 weeks post butyrate treatment, for dehydrophytosphingosine, *q* = 0.2295; for hydroxypalimotyl sphingomyelin, *q* = 0.2295; for hydroxypalimotyl sphingomyelin, *q* = 0.2295; for palimitoyl sphingomyelin, *q* = 0.037). Differences are assessed by the Welch’s two-sample *t*-test. WT (N = 6) and SOD1^G93A^ (N = 6) mice.

Our previous study has shown that bacterial butyrate delays ALS progression in SOD1^G93A^ mice by restoring the intestinal function and microbiome [4]. Here, we assessed the effect of dietary butyrate on the metabolite profiles of SOD1^G93A^ and WT mice over the course of 6 weeks. In Figure 2A, we used PCA to visually assess the similarities between the untreated and butyrate-treated groups at baseline, 3 weeks after butyrate treatment, and 6 weeks after butyrate treatment. The PCA shows moderate separation between the untreated baseline samples compared to the butyrate-treated samples at 3 weeks and 6 weeks after butyrate treatment. This suggests butyrate treatment altered the metabolic profiles of the WT and SOD1^G93A^ mice. 

In Table 2, SOD1^G93A^ mice achieved statistical significance, *p* ≤ 0.05, for a total of 262 metabolites post-butyrate treatment, suggesting significant changes due to butyrate treatment in metabolites in the SOD1^G93A^ mice. Particularly, 3 weeks after butyrate treatment, 107 metabolites were altered (of which 43 increased and 64 decreased), while 81 metabolites were altered 6 weeks after butyrate treatment (of which 32 increased and 49 decreased).

As shown in Figure 2B, an RF analysis compared metabolites between the butyrate-treated WT and SOD1^G93A^ mice, resulting in a list of the top 30 biochemicals that contribute with maximum importance. Metabolites classified as lipids such as oleoyl-linoleoyl-glycerol, lactosyl-N-palmitoyl-sphingosine, and lactosyl-N-stearoyl-spingosine had the highest degree of separation in the analysis. Additionally, other biochemical pathways showed decreasing separation, including (A) carbohydrate, (B) amino acid/protein, (C) lipid, and (D) xenobiotics metabolism. 

#### 3.2.1. Changes in Carbohydrates in Butyrate-Treated SOD1^G93A^ Mice

As shown in Figure 2C, SOD1^G93A^ mice showed a decrease in N-acetylneuraminate and N-glycolylneuraminate 3 weeks post-butyrate treatment. There is a decrease of N6-carboxymethyllysine (3 weeks post-butyrate treatment vs. baseline, 6 weeks post-butyrate treatment vs. baseline), an advanced glycation end-product that can be utilized by colonic bacteria as a source of energy [29]. These results suggest changes in carbohydrate metabolism following butyrate treatment. These differences appeared to be rectified in mice treated with butyrate. There were no time points of a statistically significant fold-change ratio for the carbohydrate metabolites listed above except for pyruvate, for which SOD1^G93A^ mice on average had a significant decrease compared to WT mice 3 weeks post-butyrate treatment. These results support the hypothesis that butyrate treatment helps rectify metabolomic alterations associated with ALS progression. Additionally, ribulonate/xylulonate/lyxonate (isomers that co-elute together) were higher in ALS mice at baseline and decreased in mutant samples in response to butyrate administration (3 weeks post-butyrate treatment vs. baseline, 6 weeks post-butyrate treatment vs. baseline). Taken together, these decreases suggest that butyrate administered to SOD1^G93A^ mice modified carbohydrate metabolism. 

#### 3.2.2. Changes in Amino Acids/Proteins in Butyrate-Treated SOD1^G93A^ Mice

Per Figure 2D, administration of butyrate resulted in lower levels of some tryptophan-related metabolites in SOD1^G93A^ mice (3 weeks post-butyrate treatment vs. baseline), which may have been caused by enhanced uptake from the gut lumen and/or increased degradation. ALS-specific changes in tryptophan catabolites were observed in SOD1^G93A^ mice. The catabolites indoleacetate, indoleacrylate, and kynurenate decreased 3 weeks after butyrate treatment compared to baseline. For WT mice, anthranilate decreased when comparing data 3 weeks after butyrate treatment vs. baseline (*p* = 0.0011, *q* = 0.0116) and 6 weeks after butyrate treatment vs. baseline (*p* = 0.0044, *q* = 0.0305). For G93A mice, no significant changes occurred over time (only comparing 6 weeks post-butyrate treatment vs. baseline, *p* = 0.0528, *q* = 0.1379). Other decreased tryptophan catabolites in phenol sulfate included indolepropionylglycine (WT mice: 3 weeks post-butyrate treatment vs. baseline, *p* = 0.0139, *q* = 0.0424; 6 weeks post-butyrate treatment vs. baseline, *p* = 0.0139, *q* = 0.0526. G93A mice: 3 weeks post-butyrate treatment vs. baseline, *p* = 0.0561, *q* = 0.1333; 6-weeks post-butyrate treatment vs. baseline, *p* = 0.0561, *q* = 0.1413), indoleacetylglycine (WT mice: 3 weeks post-butyrate treatment vs. baseline, *p* = 0.0017, *q* = 0.0123; 6 weeks post-butyrate treatment vs. baseline, *p* = 0.0017, *q* = 0.0221. G93A mice: 3 weeks post-butyrate treatment vs. baseline, *p* = 0.03781, *q* = 0.1094; 6 weeks post-butyrate treatment vs. baseline, *p* = 0.0378, *q* = 0.1217), and 3-indoxyl sulfate (WT mice: 3 weeks post-butyrate treatment vs. baseline, *p* = 0.0182, *q* = 0.0489; 6 weeks post-butyrate treatment vs. baseline, *p* = 0.0197, *q* = 0.0647). The results suggest butyrate administration alters tryptophan catabolism in a genotype-dependent manner.

#### 3.2.3. Changes in Lipids in Butyrate Treated SOD1^G93A^ Mice

Gut bacteria modulation of lipid metabolism is associated with weight gain in mice [30]. Figure 2E shows observed changes in sphingomyelins, sphingosines, and fatty acids. At 3 weeks post-butyrate treatment, palmitoyl sphingomyelin, sphingomyelin, and hydroxy palmitoyl sphingomyelin increased in SOD1^G93A^ mice, while dehydrophyotosphingosine decreased at 6 weeks post-butyrate treatment. These changes indicate fatty acid and phospholipid metabolism were altered by butyrate administration.

### 3.3. Neuroactive Metabolites Increase Significantly at Onset in ALS Mice

**Figure 3:**
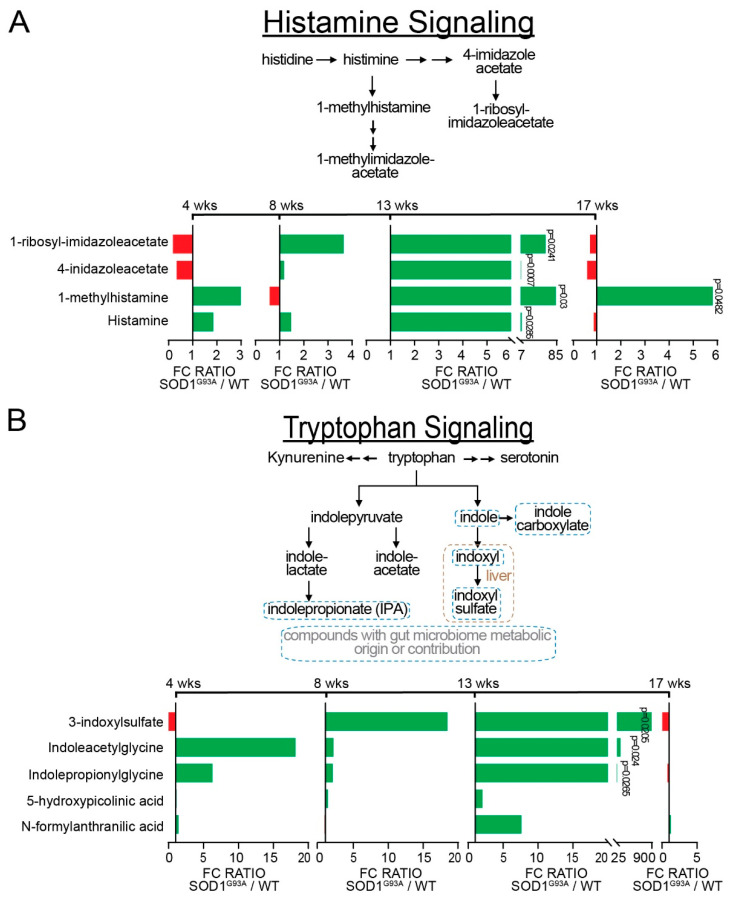
ALS altered histamine and tryptophan signaling at onset. The *p*-values and *q*-values are listed for 4, 8, 13, and 17 weeks. (**A**) Schematic of histamine signaling and longitudinal changes demonstrated by ALS mice over 17 weeks. At week 13, the histidine metabolites are 1-ribosyl-imidazoleacetate (*q* = 0.1058); 4-imidazoleacetate (*q* = 0.0532); 1-methylhistamine (*q* = 0.1133); and histamine (*q* = 0.1117). At week 17, 1-methylhistamine (*q* = 0.9941); (**B**) Schematic of tryptophan signaling, and longitudinal changes demonstrated by ALS mice over 17 weeks. At week 13, the tryptophan metabolites are 3-indoxysulfate (*q* = 0.1058); indoleacetylglycine (*q* = 0.1058); and indolepropionylglycine (*q* = 0.1091). Differences are assessed by Welch’s two-sample *t*-test. WT (N = 5) and SOD1^G93A^ (N = 5) mice.

In the CNS, the neuroactive metabolites histamine and tryptophan play a crucial role in neuroprotection against neuroinflammation in SOD1^G93A^ mice [31,32], mediate inflammation in the PNS via the production of mast cells in the gut [21], and produce commensal gut microorganisms via microbial decarboxylation of amino acids [33]. Thus, we observed changes in histamine-, tryptophan-, and glutamate-related amino acids. In Figure 3A, we show a schematic of histamine metabolism and examine changes in histaminergic signaling at different time points, using a SOD1^G93A^/WT mouse model. The histidine catabolites 1-ribosyl-imidazoleacetate, 1-methyl-4-imidazoleacetate, 1-methylhistamine, histamine, and N-acetyl-1-methylhistidine increased at 13 weeks of age in SOD1^G93A^ mice. In Figure 3B, we illustrate a schematic of the tryptophan pathway and show an increase in the tryptophan catabolites N-acetyl tryptophan, N-formylanthranilic, 5-hydroxypicolinic acid, indoleacetate, indolepropionylglycine, indoleacetylglycine, and 3-indoxyl sulfate for SOD1^G93A^ mice at 13 weeks of age. The last three metabolites had very significant increases at 13 weeks. At 17 weeks, N-acetyltryptophan and indoleacrylate decreased for SOD1^G93A^ mice. Taken together, these results suggest an alteration of neuroactive metabolites from the gut. 

### 3.4. Dietary Butyrate Treatment Altered Neuroactive Metabolites in ALS Mice over Disease Course

**Figure 4:**
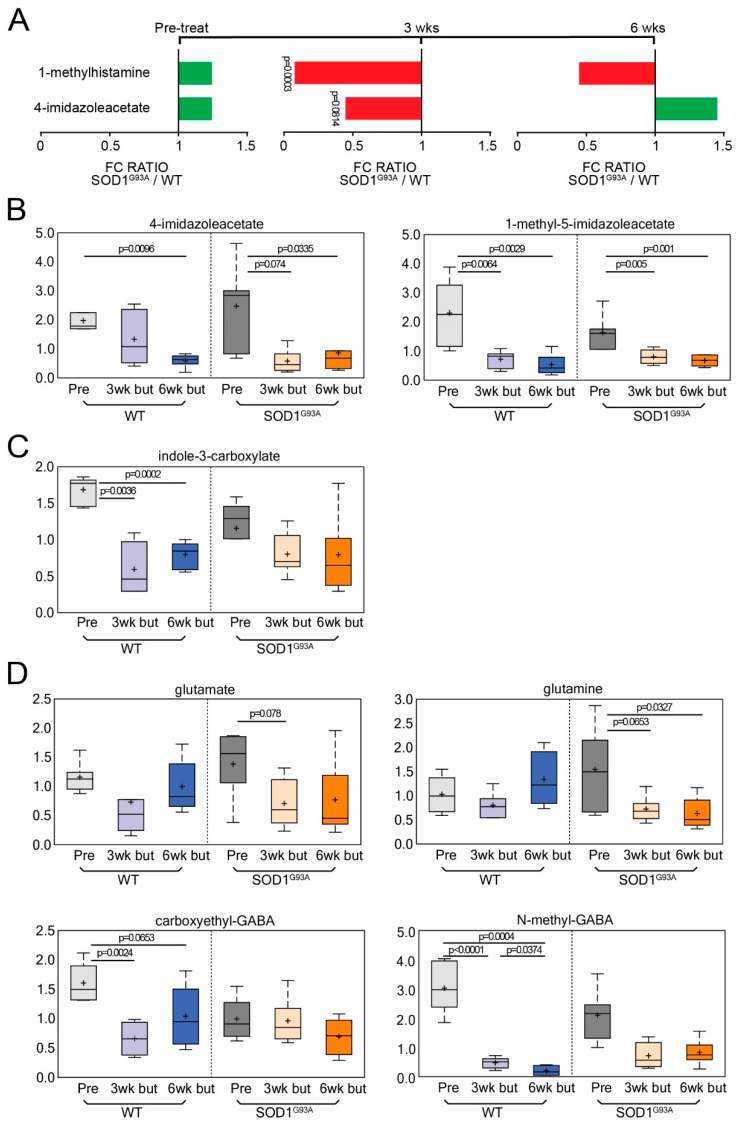
Butyrate (but) treatment altered histamine and tryptophan signaling over the course of disease. The *p*-values and *q*-values are listed for pre-treatment, 3 weeks post-butyrate treatment, and 6 weeks post-butyrate treatment. (**A**) Longitudinal changes of histamine signaling biochemical over the course of 6 weeks post-butyrate treatment. Histamine catabolites showed a significant decrease in 1-methylhistamine (*q* = 0.037) and a marginally significant decrease in 4-imidazoleacetate (q = 0.3103) at 3 weeks post-butyrate treatment (**A**). Box-plot diagrams displaying longitudinal changes of butyrate-treated animals in (**B**) 4-imidazoleacetate decreased following butyrate treatment for WT mice 6 weeks post-butyrate treatment vs. baseline: *q* = 0.0467. For G93A mice 3 weeks post-butyrate treatment vs. baseline: *q* = 0.0901, and 6 weeks post-butyrate treatment vs. baseline: *q* = 0.1215. For both WT and G93A mice: 1-methyl-5-imidazoleacetate decreased following butyrate treatment (WT: 3 weeks post-butyrate treatment vs. baseline, *q* = 0.0292; 6 weeks post-butyrate treatment vs. baseline: *q* = 0.0254. G93A: 3 weeks post-butyrate treatment vs. baseline, *q* = 0.0841; 6 weeks post-butyrate treatment vs. baseline: *q* = 0.0534). In (**C**) indole-3-carboxylate (WT: 3 weeks post-butyrate treatment vs. baseline, *q* = 0.0194; 6 weeks post-butyrate treatment vs. baseline, *q* = 0.011). (**D**) glutamate (G93A: 3 weeks post-butyrate treatment vs. baseline, *q* = 0.1612); glutamine (G93A: 3 weeks post-butyrate treatment vs. baseline, *q* = 0.1428; 6 weeks post-butyrate treatment vs. baseline, *q* = 0.1215). Due to changes in glutamine having a different time course, at 6 weeks post-butyrate treatment, WT and G93A mice reached different values of glutamine (*p* = 0.0183, *q* = 0.2617). Carboxyethyl-GABA: for WT, 3 weeks vs. baseline, *q* = 0.0149; for 6 weeks post-butyrate treatment vs. baseline, *q* = 0.1263. At baseline, there was a difference between WT and G93A mice (*p* = 0.0149, *q* = 0.2687). N-methyl-GABA: for WT (3 weeks post-butyrate treatment vs. baseline, *q* = 0.0018; 6 weeks post-butyrate treatment vs. baseline, *q* = 0.0124; 6 weeks post-butyrate treatment vs. 3 weeks post-butyrate treatment, *q* = 0.2512); for G93A (6 weeks post-butyrate treatment vs. baseline, *q* = 0.0018; 6 weeks post-butyrate treatment vs. baseline, *q* = 0.0124; 6 weeks post-butyrate treatment vs. 3 weeks post-butyrate treatment, *q* = 0.2512); 6 weeks post-butyrate treatment, there were different values between WT and SOD1^G93A^ mice (*p* = 0.0081, *q* = 0.2436). The data are presented as the fold-change (FC) ratios of the average concentrations of WT and SOD1^G93A^ mice. Differences are assessed by the Welch’s two-sample *t*-test. WT (N = 6) and SOD1^G93A^ (N = 6) mice.

We observed changes in histamine, tryptophan, and glutamate metabolites following butyrate treatment in SOD1^G93A^ mice. An analysis of histamine catabolites showed a significant decrease in the histamine catabolite 1-methylhistamine and a marginally significant decrease in 4-imidazoleacetate at 3 weeks post-butyrate treatment (Figure 4A,B). In Figure 4C, we show that the tryptophan catabolite indole-3-carboxylate decreased from baseline in WT and SOD1^G93A^ mice (baseline vs. 3 weeks post-butyrate treatment and 6 weeks post-butyrate treatment). Next, we observed changes in glutamate metabolism. γ-Aminobutyric acid is also known as GABA. In Figure 4D, glutamate, glutamine, and N-methyl-GABA decreased following butyrate treatment, while carboxyethyl-GABA only decreased for WT mice following butyrate administration. 

### 3.5. Energy-Related Metabolites Are Altered during Onset and Decreased following Butyrate Treatment in ALS Mice

**Figure 5:**
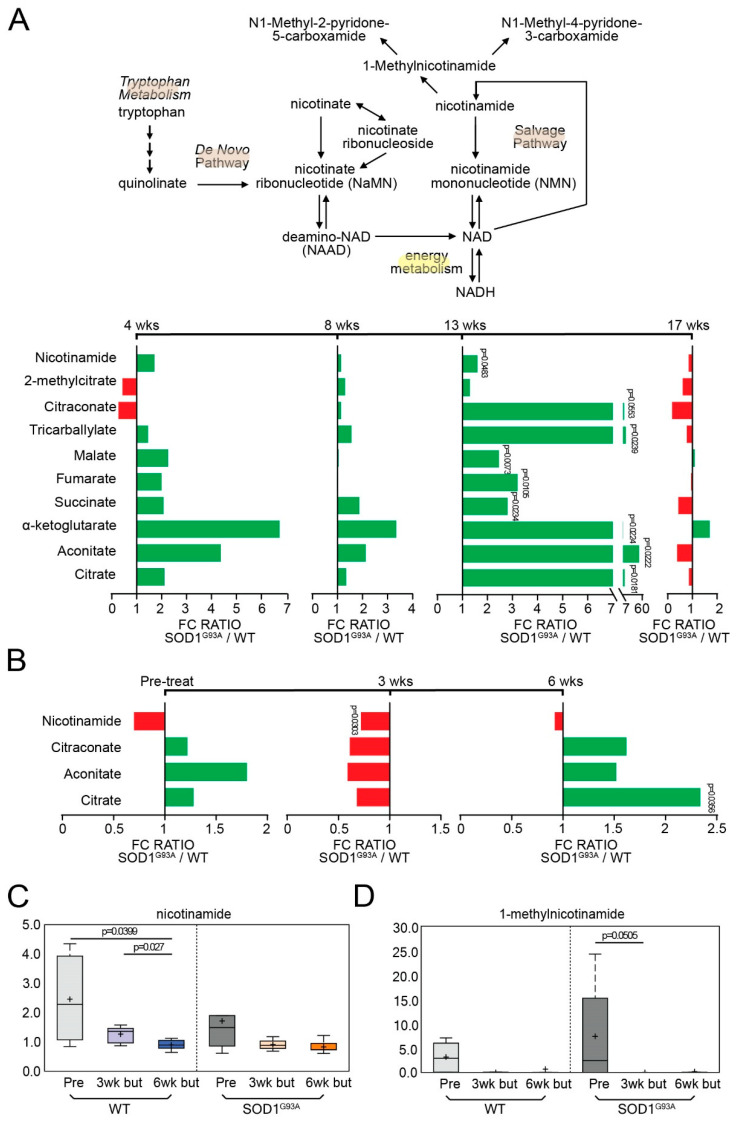
ALS altered energy-related metabolites over time and butyrate treatment altered over the course of disease. (**A**) Longitudinal changes of energy-related biochemicals in ALS mice over 17 weeks. The *p*-values for 4, 8, 13, and 17 weeks are labeled in the figures, and their according *q*-values are provided in the figure legends. Nicotinamide (week 13: *q* = 0.1298); Citraconate (week 13: *q* = 0.1372); tricarballylate (week 13: *q* = 0.1058); malate (week 13: *q* = 0.0797); fumarate (week 13: *q* = 0.0879); succinate (week 13: *q* = 0.1058); alpha-ketoglutarate (week 13: *q* = 0.1058); aconitate (week 13: *q* = 0.1058); citrate (week 13: *q* = 0.1058). (**B**) Longitudinal changes of energy-related biochemicals in butyrate-treated ALS mice over 6 weeks. The *p*-values for pre-treatment; 3 weeks post-butyrate treatment, and 6 weeks post-butyrate treatment are labeled in the figures, and their according *q*-values are provided in the figure legends. Nicotinamide (week 3 post-butyrate treatment: *q* = 0.2295); citrate (6 weeks post-butyrate treatment: *q* = 0.2729). Box plot diagrams displaying longitudinal changes of butyrate-treated animals in energy-related metabolites: (**C**) nicotinamide (WT: 6 weeks post-butyrate treatment vs. baseline, *q* = 0.0959; 6 weeks post-butyrate treatment vs. 3 weeks post-butyrate treatment, *q* = 0.2512), and (**D**) 1-methylnicotinamide (G93A: 3 weeks post-butyrate treatment vs. baseline, *q* = 0.1256). Differences are assessed by the Welch’s two-sample *t*-test. For untreated groups: WT (N = 5) and SOD1^G93A^ (N = 5) mice. For treated groups: WT (N = 6) and SOD1^G93A^ (N = 6).

Energy and mitochondrial dysfunction are involved in the pathogenesis of ALS. Animal and human studies have shown impaired mitochondrial metabolism that contributes to an increase in reactive oxygen species (ROS) [34]. We assessed the changes in mitochondria-associated metabolites during ALS. Citraconate/glutaconate, tricarballylate, alpha-ketoglutarate, aconitate, citrate, and nicotinamide significantly increased at 13 weeks of age for untreated ALS mice (Figure 5A). We investigated the effect of butyrate and found that declines in nicotinamide and 1-methylnicotinamide levels were observed in response to butyrate treatment (6 weeks post-butyrate treatment vs. baseline, WT and SOD1^G93A^). Moreover, sarcosine, dimethylglycine, and betaine were decreased in fecal samples collected from butyrate-treated mice (WT and SOD1^G93A^). These compounds are intermediates in one-carbon metabolism, which is implicated in nucleotide biosynthesis and methylation processes. Thus, these changes may potentially affect methylation patterns through modulation of substrate availability (Figure 5B). In addition, nicotinamide and 1-methylnicotinamide had decreasing trends for WT and SOD1^G93A^ mice following butyrate treatment, respectively (Figure 5C,D). These findings suggest butyrate treatment may influence energy metabolism.

### 3.6. Gamma-Glutamyl Amino Acid Metabolites Are Altered following Butyrate Treatment in ALS Mice

**Figure 6:**
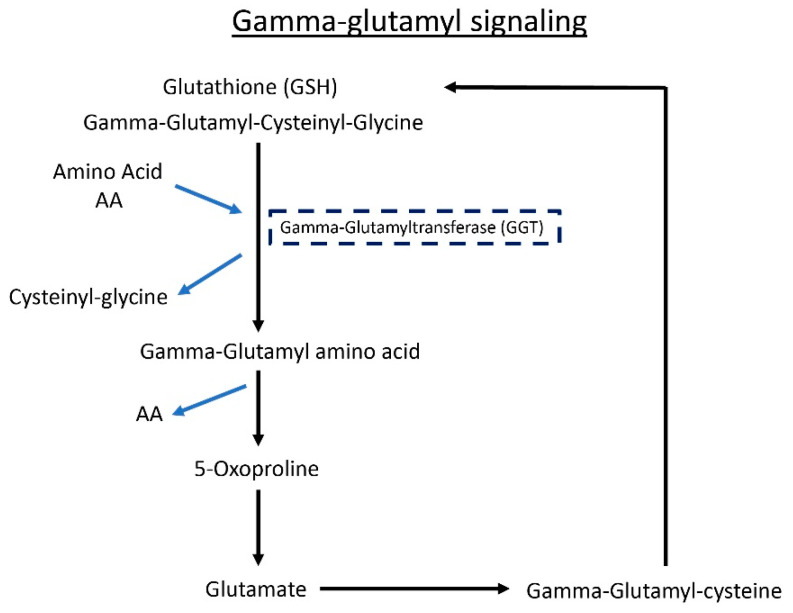
The schematic shows the GGT pathway. Detailed changes of metabolites with or without butyrate treatment are shown in Table 3.

Gamma-glutamylated forms of amino acids are generated by gamma-glutamyltransferase (GGT), an enzyme that catalyzes the transfer of the glutamyl moiety from GSH to acceptor molecules (e.g., amino acids), as shown in Fig. 6. The addition of the gamma-glutamyl moiety improves the transport of amino acids across lipid membranes. Importantly, both host cells and certain bacterial species express GGT activity. The schematic shows the GGT pathway (Figure 6). In Table 3, we show declines in fecal levels of several gamma-glutamyl amino acids in both butyrate-treated WT and SOD1^G93A^ mice, which could be interpreted as decreases in GGT activity. The light green boxes indicate a significant reduction of gamma-glutamyl amino acid metabolite levels (*p* < 0.05), while the dark green boxes show reductions that narrowly missed statistical cut-off for significance (0.05 < *p* < 0.10). It is possible that the changes observed here in response to butyrate administration may modify the neuronal function of SOD1^G93A^ mice. 

### 3.7. Short-Chain Fatty Acids (SCFA) in the WT and ALS Mice with or without Butyrate Treatment

**Figure 7:**
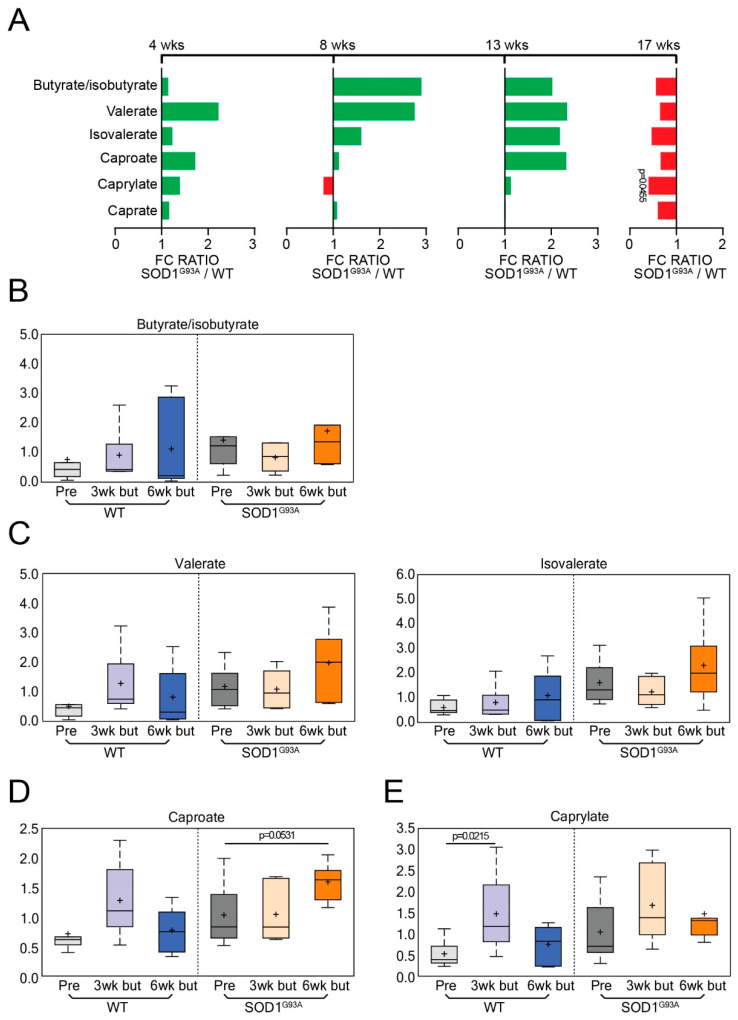
Fatty acid metabolites following butyrate administration in ALS. (**A**) Longitudinal changes of energy-related biochemicals in the WT and ALS mice over 17 weeks. The *p*-values for 4, 8, 13, and 17 weeks are labeled in the figures, and their corresponding *q*-values are provided in the figure legends. Caprylate (week 17: *q* = 0.9941). Box plot diagrams displaying longitudinal changes of butyrate-treated animals in (**B**) butyrate/isobutyrate; (**C**) valerate and isovalerate (at baseline: G93A vs. WT: *p* = 0.0111, *q* = 0.2687). (**D**) caproate (G93A: 6 weeks post-butyrate treatment vs. baseline, *q* = 0.1379; G93A vs. WT: 6 weeks post-butyrate treatment, *p* = 0.0145, *q* = 0.2617), and (**E**) caprylate (WT: 3 weeks post-butyrate treatment vs. baseline, *q* = 0.0520). Differences are assessed by the Welch’s two-sample *t*-test. For untreated groups: WT (N = 5) and SOD1^G93A^ (N = 5) mice. For treated groups: WT (N = 6) and SOD1^G93A^ (N = 6).

Butyrate treatment is vital for regulating the effects of the gut microbiome on local and systemic immunity. Thus, short-chain fatty acids are involved in the gut–brain axis [35]. We have shown that manipulating the microbiome via butyrate treatment alters the microbial community in SOD1^G93A^ animals [5]. An analysis of fatty acids showed that butyrate administration did not alter the short-chain fatty acids butyrate/isobutyrate (isobutyrate is a butyrate isomer that co-elutes together) (Figure 7A); valerate (Figure 7B); and isovalerate (Figure 7C). The medium-chain fatty acid caproate (Figure 7D) increased following long-term butyrate administration, while caprylate (Figure 7E) did not change with butyrate treatment. 

### 3.8. Butyrate Treatment Reduced Lumbar Spine IBA1 Expression, Serum IL-17, and LPS Expression in SOD1^G93A^ Mice

**Figure 8:**
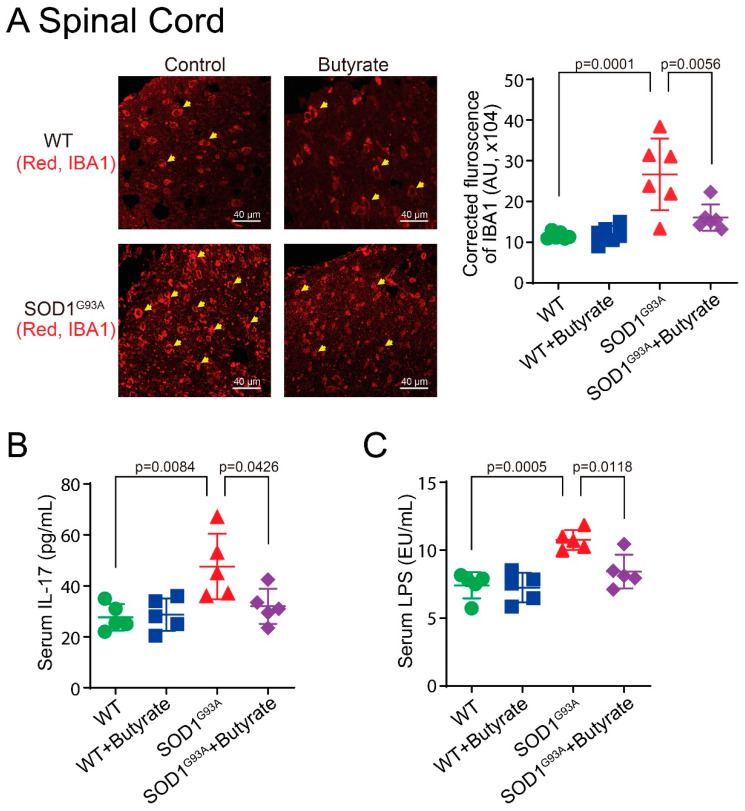
Butyrate treatment led to reduced lumbar spine IBA1 expression and reduced serum IL-17 and LPS expression in SOD1^G93A^ mice. (**A**) Decreased IBA1 expression in lumbar spine of SOD1^G93A^ mice with butyrate treatment compared with the WT group. Male or female SOD1^G93A^ mice were treated with or without 2% butyrate in drinking water starting with mice aged 9 weeks to 15 weeks. At the age of 15 weeks, mice were sacrificed, and blood and tissues were collected. Images are from a single experiment and are representative of 6 mice per group. (Data are expressed as mean ± SD. N = 6, one-way ANOVA test). (**B**) IL-17 was significantly lower in the serum of SOD1^G93A^ mice with butyrate treatment compared with the WT group. (Data are expressed as mean ± SD. N = 5, one-way ANOVA test). (**C**) LPS was significantly lower in the serum of SOD1^G93A^ mice with butyrate treatment compared with the WT group. (Data are expressed as mean ± SD. N = 5, one-way ANOVA test). The *p*-values used in the figures are the adjusted *p*-values given by the Tukey method.

Overexpression of microglia is categorized as neurotoxic and contributes to the upregulation of pro-inflammatory cytokines in ALS [36]. We have shown that manipulating the microbiome via butyrate treatment decreases some inflammatory responses in SOD1^G93A^ mice [5]. Ionized calcium binding adaptor molecule 1 (IBA1) is a microglia/macrophage-specific calcium-binding protein. Immunofluorescence analysis of microglia shows an increase in IBA1 in the lumbar spine of SOD1^G93A^ mice and significant decrease in the butyrate-treated SOD1^G93A^ mice (Figure 8A). ALS is associated with an increase in inflammatory cytokines [11]. We observed that serum IL-17 decreased in butyrate-treated SOD1^G93A^ mice compared to untreated SOD1^G93A^ mice, and serum LPS increased in SOD1^G93A^ mice and decreased in butyrate-treated SOD1^G93A^ mice (Figure 8B,C). However, the other cytokines, e.g., IL- 2, 5, 4, 6, TNFα, and interferon (INF) γ, were not significantly altered (Figure S2).

## 4. Discussion

In the current study, we investigated the longitudinal changes in fecal metabolites in SOD1^G93A^ mice with or without butyrate treatment. We also compared the changes in metabolites in age-matched WT and SOD1^G93A^ mice treated with the bacterial product butyrate. Samples were obtained from WT and SOD1^G93A^ mice at baseline, 3 weeks after butyrate treatment, and 6 weeks after butyrate treatment. In a parallel experiment, metabolic differences in feces from WT and SOD1^G93A^ mice were assessed in samples collected over the course of 13 weeks (4 weeks of age–17 weeks of age). Individual samples were loaded in an equivalent manner across the analytical platforms and statistically analyzed. We identified changes in carbohydrate levels, amino acid metabolism, and the formation of gamma-glutamyl amino acids. Understanding the metabolomics and intestinal changes will be important for the comprehension of ALS pathogenesis and identification of biomarkers for diagnosis and targets for new treatments.

These compounds in the gut can be derived from the digestion of plant hemicellulose by bacterial enzymes, and the decreases observed here could suggest that butyrate administration resulted in altered composition/activity of the intestinal microbiome. In fact, microbiota-induced changes in the excretion of plant-derived carbohydrates have been reported before in mice subjected to feeding-time restriction [37]. This decrease suggests that butyrate treatment could be mediating histamine levels through catabolites, which could influence immune responses within the gut. The gut microbiota controls neurobehavior via modulating brain insulin sensitivity and the metabolism of tryptophan, the precursor of serotonin [38]. Bacterial cells can decarboxylate histidine to form histamine using histidine decarboxylase [33].

Carbohydrates play a crucial role in shaping the composition and physiology of the gut microbiome [24]. N-acetylneuraminate and erythronate showed declines in response to butyrate treatment in samples collected from SOD1^G93A^ mice. These biochemicals can be produced by the gut microbiota from the degradation of chitin and bacterial cell walls, which suggests that butyrate in the diet affected the gut microbiome community structure. Finally, both the WT and SOD1^G93A^ groups exhibited decreases in tricarballylate, a microbial metabolite that can be derived from the TCA cycle intermediate aconitate (6 weeks post-butyrate treatment vs. baseline), and in N6-carboxymethyllysine (3 weeks post-butyrate treatment vs. baseline, 6 weeks post-butyrate treatment vs. baseline). Importantly, N6-carboxymethyllysine, an advanced glycation end product, can be utilized by colonic bacteria as source of energy [39], which further indicates differences in microbiome activity over the course of the study. Together, these results are consistent with butyrate-induced alterations in the composition of intestinal bacteria, which in turn modifies the digestion of carbohydrates in the gut. Declines in fecal levels of several pentoses in response to butyrate may be interpreted as increased absorption of carbohydrates from the gut lumen, which consequently may contribute to the prevention of weight loss in mutants.

The ALS metabolite results suggest microbial metabolites could contribute to ALS symptoms at onset via glucose metabolism. Glucose metabolic dysregulation has been implicated in ALS [25]. These fibers play a major role in shaping the gut microbiome composition and physiology. Past data indicated abnormalities in glucose metabolism within CNS of G93A mice.

Previous studies have highlighted imbalanced energy homeostasis in patients with ALS [40,41,42] and transgenic ALS-expressing mice [43,44]. Certain metabolic alterations could contribute to the energy imbalance in ALS. Human studies have consistently found changes in glutamate metabolite levels by assessing the serum. One study examined the metabolite profile of serum from patients with ALS via ^1^H NMR spectroscopy and found an increase in glutamate yet a reduction of glutamine levels, which indicates an imbalance in the glutamate–glutamine conversion cycle [45]. A different study used ^1^H NMR analysis to examine 17 metabolites in patients with ALS and found a reduction of acetate and increases in acetone, pyruvate, and ascorbate concentrations [46]. When comparing CSF metabolism between SALS and FALS, the results showed FALS had reduced glutamate and glutamine levels [47]. The alteration of glutamate in ALS supports the hypothesis of glutamate toxicity. However, other metabolites are also altered in ALS. An untargeted metabolomics study found post-diagnosis patients with ALS have an increased benzoate metabolism, ceramide, creatine metabolism, and fatty acid metabolism, which suggests emerging pathways contribute to ALS [48].

Animal studies indicate alterations in the cerebrospinal fluid or the serum levels. A 2019 study examined the metabolites in the lumbar and thoracic spinal cord of two ALS mouse models and found alterations in lipid- and energy-related metabolites [49]. Amino acid metabolites change during ALS; Bame et al. (2014) [50] found amino acid profiles of ALS mice change between the ages of 50 and 70 days. Additionally, the amino acids cysteine, methionine, glycine, sarcosine, phosphoserine, and phosphoethanolamine were decreased in SOD1^G93A^ mice compared to WT. These metabolites are involved in the urea cycle, neuron excitation, and methylation and related to the transsulfuration pathway.

Our data indicate the role of metabolites in neurophysiology. SOD1^G93A^ mice have increased kynurenine, a pathway associated with inflammatory neurological disorders [51]. SOD1^G93A^ mice displayed significant downregulation of quinolinate and tocopherol pathway-derived metabolites and an increase in nicotinamide. Quinolinic acid acts as a neurotoxin, proinflammatory mediator, and prooxidant molecule [52]. The gut microbiota utilizes catabolism to ferment proteolytic amino acids as an energy source [53]. The gut microbiota produces these metabolites via colonic fermentation of dietary fibers [54]. Tryptophan is an essential amino acid that cannot be produced by the human body and must be obtained through the diet. Tryptophan catabolites are converted by various bacterial species [26]. Defects in the tryptophan pathway might be associated with the development of ALS. A previous study also suggested that the kynurenine pathway metabolites could be biomarkers of ALS [55].

Because untargeted global metabolomic profiling cannot distinguish between butyrate and isobutyrate, a quantitative SCFA panel is better suited to quantify butyrate in fecal samples. The administration of the microbial metabolite butyrate did not result in significant increases in fecal levels of butyrate/isobutyrate (4:0) (these are isomers that co-elute together). This could be due to bacterial metabolism of this SCFA and/or avid uptake of butyrate from the intestines. Biochemicals that showed positive correlation with fecal butyrate/isobutyrate levels included both short-chain fatty acids (e.g., valerate (5:0) and isovalerate (C5)) and medium-chain fatty acids (e.g., caproate (6:0) and caprylate (8:0)). It is possible that butyrate supplementation is directly linked to these changes, and after incorporation into bacterial pathways, butyrate is elongated. Alternatively, these changes may represent an indirect effect of butyrate treatment through the modification of the bacterial community structure and subsequent enrichment in these fatty acids.

The intestinal microbiome generates several metabolites from carbohydrates, amino acids, and other dietary sources. Several of these metabolites can bind to cellular receptors, which could influence host functions [33]. We identified microbes within the fecal microbiome of SOD1^G93A^ mice that are altered with butyrate treatment: the bacteria *Staphylococcus. Sp. UAsDu23* decreased, and *Lachnospiraceae bacterium A4* increased [5]. Bacteria in the genus staphylococcus are pathogens of humans and other mammals [56]. The *Lachnospiraceae* is involved in immune response. N-acetyl−1−methylhistidine is known as an L-histidine derivative and an N-acetyl-L-amino acid. It has a role as a human blood serum metabolite. A previous study has identified four potential disease-associated paths, e.g., incident chronic kidney disease, and TREH with trehalose and incident diabetes have direct longitudinal predictive relationships with N-acetyl-1-methylhistidine [57]. Our study is the first to show the potential link of N-acetyl-1-methylhistidine through the amino acids/protein pathway and microbiome with ALS. Deoxycholic acid 3-sulfate, also known as 3-sulfodeoxycholic acid, is a sulfate derivative of the bile acid (BA) deoxycholic acid and an end product of intestinal microbial metabolism. Its changes were positively correlated with organisms of the bacterial taxa *Bacteroides acidifaciens JCM 10556*, *Alloprevotella*, and *Lachnospiraceae bacterium A4*, but negatively correlated with the *Clostridiales* vadinBB60 group (data not shown). It is reported that *Akkermansia muciniphila* ameliorates, whereas *Ruminococcus torques* and *Parabacteroides distasonis* exacerbate, the symptoms of ALS in the SOD1^G93A^ mice [18]. Another metabolite identified to be associated with ALS is nicotinamide (vitamin B6). However, in vivo treatment with nicotinamide resulted in a non-significant trend of improved survival in SOD1^G93A^ mice [18]. This suggests more metabolites may contribute directly to ALS or have syngenetic roles to restore the progression of ALS. Further investigation of the microbial metabolites and microbiome is needed to uncover the effects and mechanisms of specific bacterial species involved in metabolic production in vivo.

Understanding metabolism in ALS through the gut microbiome is a growing field. We have shown the beneficial role of butyrate in treating intestinal defects [4]. A recent study has shown a direct impact of a gut microorganism on improving nicotinamide levels in ALS mice [18]. Human studies also yield some similar findings. A human study used 50 patients with ALS and 50 controls and found that the gut microbiome changes during the disease course [58]. A more recent study with 20 ALS participants found alterations in the gut microbiome and metabolism [59]. The microbiota controls neurobehavior via modulating brain insulin sensitivity and the metabolism of tryptophan, the precursor of serotonin [38]. The role of the gut–neuron–microbiome axis needs further investigation in future research.

There are limited therapies to treat patients with ALS. Our current study tried to explore the butyrate treatment for ALS by targeting metabolites. Paganoni et al. reported a phase 2 randomized, placebo-controlled trial involving 137 patients with ALS, 89 of whom were treated with a combination of sodium phenylbutyrate and taurursodiol [60]. This combination has been shown to reduce neuron death and features of neurodegenerative diseases (including ALS) in preclinical models. This small trial, which treated patients for 24 weeks, found a modest reduction in functional decline in patients receiving the combination therapy [60]. This is an encouraging finding, and larger trials testing more patients over a longer period are expected. The related changes of the human microbiome and metabolites will help us to understand the efficacy and mechanism of the combination therapy.

Serum IL-17 and LPS were significantly reduced in the butyrate-treated SOD1^G93A^ mice, but this was not the case for the other inflammatory cytokines (e.g., IL- 2, 5, 4, 6, TNFα, and INFγ). This suggests specificity of IL17 and LPS in the progression of ALS. Increased serum LPS is reported in sporadic ALS (SALS) patients [10,61]. SALS and FALS may have different initiating causes that lead to a mechanistically similar neurodegenerative pathway forms of ALS and clinical phenotypes. Patients with ALS have elevated intestinal inflammation and dysbiosis [17,62]. Some clinical studies have indicated intestinal abnormalities in patients with ALS [63,64,65]. We summarized these observations of ALS GI issues in a recent review [66]. Integrated “omic” biomarkers of the microbiome and metabolites will help with the ALS diagnosis. We are aware that the top biochemicals between treatments and over the disease course currently identified by machine learning techniques need be confirmed in the future when large databases with similar studies in ALS are available. We also notice that the identified metabolites in the current report need further validation and functional studies in vivo. Understanding the inter-organ communications link among metabolites, inflammation, and ALS progression will provide novel insights into the potential to use of metabolites as ALS hallmarks and for treatment.

In conclusion, our study provides insights into the fundamental roles of metabolites in ALS. The results from this global metabolomic study assessing the metabolic effects of butyrate administration on the biochemical profiles of feces collected from WT and SOD1^G93A^ mice, together with longitudinally collected samples from untreated WT and mutant animals, differed in a number of metabolic readouts, including changes in carbohydrate levels, amino acid metabolism, and the formation of gamma-glutamyl amino acids. Differences in carbohydrates after butyrate treatment could be due to altered digestion and/or absorption of carbohydrates from the gut, which may be a consequence of changes in the intestinal microbiota. Shifts in several microbially contributed catabolites of aromatic amino acids are in agreement with butyrate-induced changes in the composition of the gut microbiome. Declines in gamma-glutamyl amino acids in feces may stem from differential expression of GGT in response to butyrate administration. Due to the signaling nature of amino acid-derived metabolites, these changes could indicate changes in inflammation (e.g., histamine) and contribute to differences in systemic levels of neurotransmitters (e.g., GABA, glutamate).

As a path forward, assaying plasma and brain samples from the butyrate experiment could allow the characterization of systemic changes occurring after the treatment. Given that butyrate administration resulted in changes in NAD metabolism and one-carbon metabolism, which can influence methylation processes, epigenetic markers in response to butyrate might provide additional functional explanations for the protective effects of this compound in ALS. Novel therapeutic methods to target metabolites will improve the life quality of patients with ALS.

## Figures and Tables

**Table 1:** ALS mice experienced metabolic alterations over the course of disease compared to WT.

Statistical Comparisons				
Welch’s Two-Sample *t*-Test	SOD1^G93A^/WT			
	4 Week	8 Week	13 Week	17 Week
**Total biochemicals *p* ≤ 0.05**	61	7	180	10
**Biochemicals (↑↓)**	49 | 12	5 | 2	175 | 5	6 | 4

**Table 2:** Butyrate (But) treatment contributes to the altered profile of metabolites in ALS mice, compared to WT mice.

Statistical Comparisons			
Welch’s Two-Sample *t*-Test	SOD1^G93A^/WT		
	Baseline	3 Week Post Butyrate Treatment	6 Week Post Butyrate Treatment
Total biochemicals *p* ≤ 0.05	74	107	81
Biochemicals (↑↓)	61 | 13	43 | 64	32 | 49

**Table 3:** Butyrate treatment causes shifts in the gamma glutamyl amino acid profile in ALS compared to WT mice.

	WT		G93A	
Biochemical Name	3 Week Post-Butyrate Treatment /Baseline	6 Week Post-Butyrate Treatment /Baseline	3 Week Post-Butyrate Treatment /Baseline	6 Week Post-Butyrate Treatment /Baseline
Gamma-glutamylalanine	1.34	0.59	0.51	0.92
Gamma-glutamylglutamate	0.49	0.33	0.26	0.43
Gamma-glutamylglutamine	0.79	0.78	0.30	0.42
Gamma-glutamylglycine	0.86	0.49	0.39	0.69
Gamma-glutamylhistidine	0.48	0.50	0.33	0.41
Gamma-glutamylisoleucine	0.81	0.61	0.37	0.61
Gamma-glutamylleucine	1.30	0.91	0.36	0.64
Gamma-glutamyl-apha-lysine	0.90	0.60	0.37	0.58
Gamma-glutamyl-epsilon-lysine	1.39	1.84	0.37	0.46
Gamma-glutamylmethionine	1.43	1.16	0.25	0.60
Gamma-glutamylphenyalanine	0.88	0.62	0.30	0.57
Gamma-glutamylthreonine	0.98	0.71	0.35	0.69
Gamma-glutamyltryosine	0.73	0.72	0.33	0.75
Gamma-glutamylvaline	1.20	0.74	0.32	0.59
Gamma-glutamylserine	0.57	0.56	0.44	0.69

## Data Availability

The data presented in this study are available in article and supplementary material.

## Supplementary Materials

The following are available online at https://www.mdpi.com/article/10.3390/metabo12121253/s1, Figure S1: Preparation of client-specific technical replicates. Figure S2: After butyrate treatment, there are no changes of expression of IL-2, IL-4, IL-5, IL-6, TNF-α, and IFN-γ in the serum of both WT and SOD1G93A mice.
