# Supplementary material for: Target Metabolites to Slow Down Progression of Amyotrophic Lateral Sclerosis in Mice
Source: Metabolites. 2022 Dec 12;12(12):1253. doi: 10.3390/metabo12121253 (PMC9784240; doi:10.3390/metabo12121253)
Supplement: Supplementary file 1 [file metabolites-12-01253-s001.zip › metabolites-2071115-supplementary.pdf]

Appendix/Supplementary

Figure S1. **Preparation of technical replicates.** A small aliquot of each sample (colored cylinders) is pooled to create a CMTRX technical replicate sample (multi-colored cylinder), which is then injected periodically throughout the platform run. Variability among consistently detected biochemicals can be used to calculate an estimate of overall process and platform variability.

S1

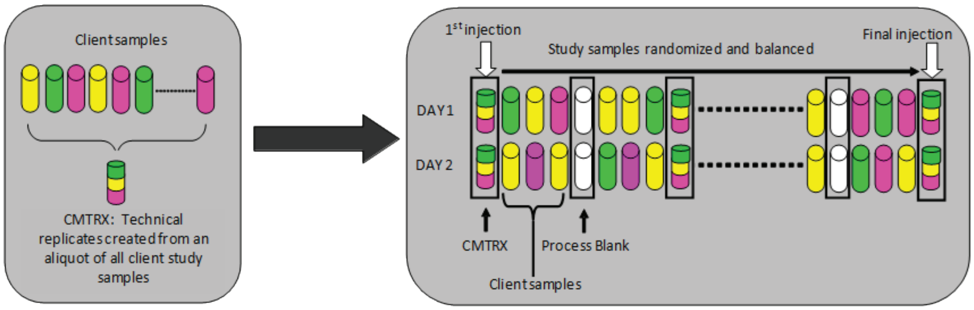
Client samples
CMTRX: Technical replicates created from an aliquot of all client study samples
1st injection
Study samples randomized and balanced
Final injection
DAY 1
DAY 2
CMTRX
Process Blank
Client samples

Figure S2. IL-2, IL-4, IL-5, IL-6, TNF-α, and IFN-γ in the serum of both WT and SOD1^{G93A} mice. (A) IL-2 **(B)** IL-4 **(C)** IL-5 **(D)** IL-6 **(E)** TNF-α and **(F)** IFN-γ levels in the serum of WT and SOD1^{G93A} mice without or with butyrate treatment. (Data are expressed as mean ± SD. n = 5, one-way ANOVA test).

S2

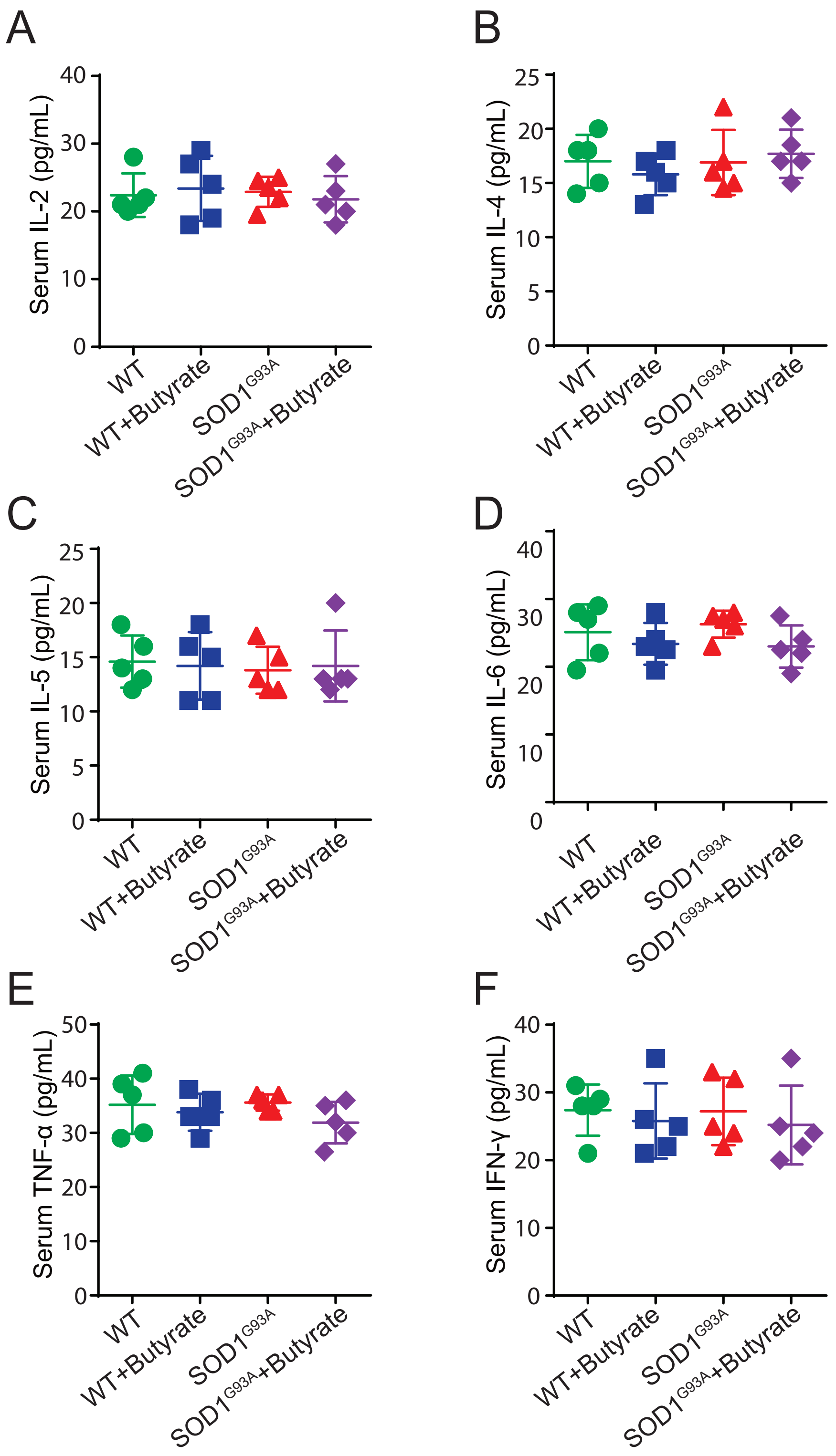
A
Serum IL-2 (pg/mL)
B
Serum IL-4 (pg/mL)
C
Serum IL-5 (pg/mL)
D
Serum IL-6 (pg/mL)
E
Serum TNF-α (pg/mL)
F
Serum IFN-γ (pg/mL)
WT
WT+Butyrate
SOD1G93A
SOD1G93A+Butyrate
